# Gout Due to Tacrolimus in a Liver Transplant Recipient

**DOI:** 10.7759/cureus.4247

**Published:** 2019-03-13

**Authors:** Summia Matin Afridi, Srikar Reddy, Ahmad Raja, Akriti G Jain

**Affiliations:** 1 Internal Medicine, Florida Hospital, Orlando, USA; 2 Internal Medicine, University of Central Florida, Orlando, USA; 3 Internal Medicine, Presence Saint Francis Hospital, Evanston, USA

**Keywords:** gout, hyperuricemia, liver transplant, tacrolimus, rheumatology

## Abstract

Patients taking tacrolimus have an increased predisposition to hyperuricemia. Although literature has widely established the risk of gout in patients taking cyclosporine, the widespread use of tacrolimus in patients following liver transplantation necessitates further investigation into the potential connection between the drug’s use and gout. Moreover, hyperuricemia in the context of liver transplants is associated with increased morbidities and mortalities. We describe a case of gout in a liver transplant patient taking the calcineurin inhibitor tacrolimus.

## Introduction

Gout typically manifests earlier in life in men than women and is rare in childhood. Non-modifiable risk factors for gout include age, gender and ethnicity; modifiable risk factors include obesity, diabetes mellitus, chronic kidney disease, and hypertension [1]. Within renal transplant patients, the relationship between gout and cyclosporine is well established [2]. Following renal transplants, there is belief that uric acid secretion can decrease; cyclosporine exacerbates these uric acid levels due to the side effects of hyperuricemia and reduced glomerular filtration rate (GFR). We present a case of newly diagnosed gout in a liver transplant patient taking tacrolimus.

## Case presentation

A 60-year-old gentleman with past medical history of liver transplant five years ago presented to the hospital with acute onset of right-sided knee pain. For his immunosuppressive regimen, he took 2 mg/day of tacrolimus. His complete medication history was reviewed and no significant drug-drug interactions were found. His social history was negative for excessive alcohol use and high-protein diet. His physical examination was significant for right knee warmth, swelling, and erythema with tenderness upon palpation.

Labs indicated normal white blood cell count, normal creatinine at 0.81 mg/dl, tacrolimus at 9.3 ng/ml, uric acid at 6.1 mg/dl, and elevated C-reactive protein at 18.1 mg/L. Synovial fluid analysis showed 27,000 nucleated cells with differential of >90% neutrophils and 1+ monosodium urate crystals (Table 1). Fluid cultures were negative and ruled out septic arthritis. This patient was diagnosed with acute gouty arthritis, and the patient was administered colchicine for three days. His tacrolimus dosage was decreased from 2 mg/day to 1 mg/day. With treatment, the patient’s symptoms resolved, and he was continued on the adjusted dose of tacrolimus with outpatient follow-up.

**Table 1:** Synovial Fluid Analysis. RBC: Red Blood Cell

Characteristics	Findings
Color	Yellow
Fl Nucleated Cells	27,000
Fl RBCs	333
Neutrophils	93
Lymphocytes	0
Monocytes	7
Microscopy	Intra-cellular Monosodium Urate Crystals 1+
pH	7.8
Glucose	122

## Discussion

For tacrolimus, the effect on uric acid levels is not as well established compared to cyclosporine’s effect [3]. Hyperuricemia has been reported in patients taking tacrolimus, but there have been only a few reported cases of gout [4, 5]. The reason for the discrepancy between cyclosporine-induced and tacrolimus-induced gout may be that cyclosporine can promote increased uric acid reabsorption in the proximal tubules and decreased GFR following afferent arteriole vasoconstriction, whereas tacrolimus is only known to reduce the excretion of uric acid [6]. Even though this particular patient possessed risk factors for gout such as male gender, his acute gouty attack may have been precipitated by the use of tacrolimus for his immunosuppressive regimen following his liver transplant. Hyperuricemia can be seen in 14-47% of liver transplant patients, predominantly due to accompanying decreased renal function [7].

In liver transplant patients, tacrolimus has emerged as the go-to maintenance regimen over cyclosporine due to data indicating increased patient and graft survival and decreased acute rejection [8]. Therapeutic levels of tacrolimus remain controversial. They need to be individually catered to patients and their specific comorbidities and functional status. Current guidelines indicate the following: in the first four to six weeks following a liver transplant, the trough levels of 10-15 ng/ml are recommended and 5-10 ng/ml thereafter to maintain a balance between nephrotoxicity and acute rejection [9]. In the context of our patient (tacrolimus level at 9.3 ng/ml), his tacrolimus levels were on the upper range of target trough levels and may have been significant enough to cause tubular dysfunction. Since tacrolimus undergoes liver metabolism, the elevated tacrolimus levels in a patient with liver transplant combined with noncompliance with outpatient follow-up may have contributed to hyperuricemia and the development of gout.

## Conclusions

Every clinician should be aware of potential side effects of calcineurin inhibitors such as cyclosporine and tacrolimus. Their effects should be monitored during initial hospitalization, and expert opinion should be sought for dose adjustments. Also, the patients should be advised about the importance of regular outpatient follow-up to monitor drug levels and avoid the potential of drug-induced toxicities.
